# Antimicrobial Activity of Tea and Agarwood Leaf Extracts Against Multidrug-Resistant Microbes

**DOI:** 10.1155/bmri/5595575

**Published:** 2024-12-19

**Authors:** Shah Rucksana Akhter Urme, Syeda Fahmida Ahmed, Md Abdus Shukur Imran, Mst Rubaiat Nazneen Akhand, Mohammad Mehedi Hasan Khan

**Affiliations:** ^1^Department of Biochemistry & Chemistry, Sylhet Agricultural University, Sylhet, Bangladesh; ^2^Department of Animal and Fish Biotechnology, Sylhet Agricultural University, Sylhet, Bangladesh; ^3^Department of Pharmaceutical and Industrial Biotechnology, Sylhet Agricultural University, Sylhet, Bangladesh

**Keywords:** agarwood leaves, antimicrobial activity, phytochemicals, synergistic activity, tea

## Abstract

Emerging multidrug-resistant (MDR) strains are the main challenges to the progression of new drug discovery. To diminish infectious disease–causing pathogens, new antibiotics are required while the drying pipeline of potent antibiotics is adding to the severity. Plant secondary metabolites or phytochemicals including alkaloids, phenols, flavonoids, and terpenes have successfully demonstrated their inhibitory potential against the drug-resistant pathogens. In quest of potential phytochemicals, we selected tea (*Camellia sinensis*) and agarwood (*Aquilaria malaccensis*) leaves for antimicrobial activity. Fresh tea leaves were collected in three varieties, namely, BT-6, BT-7, and BT-8, including green tea (nonfermented tea), black tea (fully fermented tea), and agarwood leaves collected from Sylhet region of Bangladesh. This study is aimed at analyzing the phytochemical constituency and antimicrobial activity of tea and agarwood leaf extracts and analyzing if there is a combined effect or synergistic activity against multidrug-resistant pathogens. The antimicrobial activity of tea and agarwood leaf extracts was analyzed against MDR pathogenic bacteria and fungus. Qualitative and quantitative phytochemical constituency profiling of these six leaf extracts was evaluated, and preliminary screening exhibited that most of the leaves contained diverse groups of metabolites (alkaloids, tannin, flavonoids, glycosides, saponins, etc.). The highest amounts of TPC (total phenolic content) (110.16 ± 0.48 *μ*g/mg) were found in BT-7 in ethanol extracts, and BT-8 in methanol extracts possessed the highest (128.1 ± 0.43 *μ*g/mg) TFC (total flavonoid content). Notably, green tea showed remarkable results in TPC and TFC. In antioxidant scavenging activity, BT-7 and green tea showed significant IC_50_ values which were 13.23 and 20.75 mg/mL, respectively. In antimicrobial assays, both 50 *μ*L of each tea and agarwood leaf extract antimicrobial activities were examined against 50 *μ*L of each bacterial and fungal culture. In synergistic activity, 50 *μ*L of each type of leaf extracts was poured over the commercial antibiotics to evaluate their synergism, additive, or antagonism activity against the multidrug-resistant pathogens. In the antimicrobial activity test, green tea showed a maximum diameter (22.0 ± 1.1 mm) zone of inhibition against *Klebsiella pneumoniae* whereas BT-8 showed 22.0 ± 2.5 mm against *Pseudomonas aeruginosa*. Indeed, fresh tea BT-6 and BT-7 both showed remarkable zone of inhibition against the selected microbes including Gram-negative and Gram-positive bacteria. Besides, leaf extract also showed antimicrobial activity against pathogenic fungus *Mucor circinelloides*. Aiming to increase antibiotic resistance efficacy, synergistic activities were evaluated among leaf extracts and antibiotics against the selected pathogens where synergism, antagonism, and additive results were noted. Combination of BT-8 extracts with antibiotics (ceftiofur) showed the highest synergism nearly 36 mm of the zone of inhibition against *Escherichia coli*. Additionally, green tea with gentamicin and erythromycin also showed remarkable synergism 35 and 33 mm against *Mucor circinelloides* and *E. coli*, respectively. Tea and agarwood leaves grown in Bangladesh possess high antioxidant activity, promising antibacterial and antifungal activity, thus might provide a potential source for drug discovery.

## 1. Introduction

Antibiotics have extensively been used to improve public health worldwide while antibiotic resistance is emerging as a serious threat to human healthcare globally. The increasing rate of drug-resistant pathogenic microorganisms, coupled with the decreasing rate of new antibiotic development, leads to a threat to the healthcare system. Moreover, the adverse impact of commercial antibiotics on both human and farm recently raised public awareness and concern. This alarming situation requires alternative therapeutic agents that are increasing due to decreasing drug activity or microbial susceptibility. It is discussed long ago that plants synthesize phytochemicals, which are metabolites and a variety of secondary metabolites needed for defense mechanisms and environmental stress [1]. From ancient times, the use of antimicrobial compounds obtained from therapeutic agents including plant-derived metabolites for reducing pathogenic bacteria encourages an increase in the demand for medicinal plants for treatment, such as tea components [2]. On the other hand, infectious diseases are one of the biggest risks to the world, causing more than 50 million deaths annually, and the number of food-related illnesses brought on by pathogens is steadily rising [2, 3]. Foodborne illnesses are strikingly associated to pathogenic bacteria like *Escherichia coli*, *Salmonella enterica*, *Bacillus cereus*, *Campylobacter jejuni*, *Listeria monocytogenes*, and *Staphylococcus aureus* [2]. In addition to dangerous bacteria, fungi, including *Mucor circinelloides*, are also frequently responsible for deadly illnesses. Moreover, antibiotic or microbial drug resistance leads to the worst situation. Antibiotic resistance and main reasons behind the chronic and repeated infections are intense and inappropriate use of antibiotics, irrational farm animal antibiotic use, limitations of drugs, inadequate investigation, and miserable healthcare standards [4, 5]. In this time of global crises, researchers and experts are working to find a comprehensive alternative such as the use of therapeutic plant chemicals [6]. In the case of antibiotic resistance, researchers explained that natural remedies from plants are the most preferable solution as they are less expensive and have the least amount of side effects or diseases [7, 8]. Plant polyphenol bioactive compounds contain therapeutic importance [9]. Researchers have found phenolics and flavonoid complexes together with saponins and alkaloids have a proven record as antimicrobial agents in numerous plants [10, 11]. However, the regional variance of the plant growing, for example tea, might affect genetic variation, variety quality, and interactions with the environment [12]. Tea is a leaf infusion that has been enjoyed for ages as a beverage and is highly prized for its therapeutic benefits. There are three different kinds of tea dependent on the method in addition to freshly cut leaves: green and white teas are not fermented, while red and oolong teas are just partially fermented and black tea (BT) is fermented [13]. The polyphenols and valuable bioactive compounds such as alkaloids, flavonoids, steroids, phenols, and terpenoids are present in green tea (GT) leaves that are responsible for medicine development [14]. The flavonoids, or catechins, are natural polyphenols found in GT that play an active role in antioxidant functions such the neutralization of free radicals produced during the metabolic process [15]. BT leaves and buds are fermented or oxidized after they have been dried [16]. Agarwood is now used in more than only commercial aromatic items; it also contributes to pharmaceuticals with a variety of bioactivities, including analgesic, antipyretic, antihyperglycemic, antiasthmatic, antibacterial, face and skin treatments, and malaria medications [17, 18]. Reports suggest that several amounts of phytochemical compounds isolated from plants have potential application in the field of development of novel drugs to inhibit the growth of bacterial and fungal pathogens [19]. There has been extensive research on medicinal plants' phytochemical and antimicrobial activity, but often, tea and agarwood leaves and their antimicrobial and synergistic activity against bacteria and fungus are overlooked. However, extraction yields, solvent type, and the content of bioactive compounds (phenolics, alkaloids, flavonoids, and terpenoids) varied among the extracts. In this study, tea (*Camellia sinensis*) and agarwood (*Aquilaria malaccensis*) were selected based on their popularity as widely consumed beverage and commercial economic importance; additionally, these plants have been recognized for traditional uses in pharmacological sectors too. This study is aimed at analyzing tea and agarwood leaf extract antimicrobial activity against pathogenic multidrug-resistant microbes.

## 2. Methods

### 2.1. Collection of Pathogenic Microbes

A total of five pathogenic bacteria: *E. coli*, *Pseudomonas aeruginosa*, *Klebsiella pneumoniae*, *Salmonella* spp., and one Gram-positive *S. aureus*, were collected from the Department of Plant and Environmental Biotechnology. One pathogenic fungus named *Mucor circinelloides* was collected from the Department of Biochemistry and Chemistry, Sylhet Agricultural University, Sylhet, Bangladesh. A total of five commercial antibiotics were selected according to their class, generation, and mode of action. These are vancomycin (Van) (30 *μ*g), gentamicin (Gen) (10 *μ*g), erythromycin (E) (30 *μ*g), ceftiofur (CTR) (10 *μ*g), and amoxicillin (Amx) (10 *μ*g).

### 2.2. Collection of Plant Leaves

In this study, plant samples of tea and agarwood leaves were collected from Sylhet, Bangladesh. Three types of tea were collected from Sylhet region in particular, fresh/unprocessed tea variety from BT-7 from Moulvibazar: 24.4859° N, 91.7765° E; BT-6 from Sylhet: 24.9048° N, 91.8600° E; and BT-8 from Habiganj: 24.3750° N, 91.4167 °E. Both fermented (BT) and nonfermented (GT) tea were collected from the local market (company name: Ispahani Tea) in Sylhet.

### 2.3. Extraction of Phytochemicals

Tea (BT-6, BT-7, BT-8, GT, and BT) and agarwood leaves were washed separately in running clean water for 10 min to remove undesirable debris and dust. To extract secondary metabolites from leaves, a solvent extraction process was followed [20, 21]. Samples were transported to the laboratory for drying 40 ± 3°C for 36 h where above 45°C may damage the phytochemicals. The electronic hot dryer oven (Biobase Company) was used to dry leaves which were powdered using an electronic blender and kept in different labeled plastic boxes for further experiments. Three solvents: methanol, ethanol, and chloroform, were used for each type of tea and agarwood leaf phytochemical extractions. Five grams each of powdered leaf samples was soaked in a conical flask containing 80 mL of the methanol, ethanol, and chloroform solvents, and flasks were covered with aluminum foil. The liquid phase was separated from the residues by filtering with Whatman No. 1 filter paper, and then, it was kept for 7 days at room temperature for quick extraction of phytochemicals. The organic solvents were removed by evaporation using a hot air oven at 40°C. After the removal of the organic solvent, residues were resuspended on DMSO solution at a concentration of 1 mg/mL and stored in a refrigerator at −4°C until further application. The crude extracts were subjected to qualitative and quantitative test analysis and antimicrobial activities against different bacterial strains and one fungal strain.

### 2.4. Qualitative Analysis of Phytochemical Constituents

Preliminary qualitative phytochemical analysis was carried out to identify the secondary metabolites present in the solvent (methanol 0.1 mg/mL). The tests were analyzed to find the presence of the active chemical constituents, in particular alkaloids, glycosides, terpenoids and steroids, flavonoids, and tannin. For the alkaloid test, methanol leaf extracts were dried, and the residue was heated on a boiling water bath with 2% hydrochloric acid in six separated test tubes. After cooling, the mixture was filtered and treated with a few drops of 5% sodium hydroxide solution. The samples were then observed for the presence of turbidity or yellow precipitation for indication of alkaloids followed by Wagner's test [22].

In the glycoside test, 0.5 g of leaf extracts was mixed with 2 mL of glacial acetic acid; after that, few drops of ferric chloride and concentrated sulfuric acid were added and observed for a reddish–brown coloration at the junction of two layers and the bluish green color in the upper layer for positive result [23]. The terpenoid test is where each 4 mg of extracts was treated with 0.5 mL of acetic anhydride and 0.5 mL of chloroform. After that, a concentrated solution of sulfuric acid was added slowly and red–violet color was observed for the presence of terpenoid [24]. Like in the terpenoid test, 4 mg of extract was treated with 0.5 mL of acetic anhydride and 0.5 mL of chloroform. Then, a concentrated solution of sulfuric acid was added slowly and green–bluish color was observed for the presence of steroids [24]. According to the NaOH test, each 1 g methanol extract dissolved in methanol for solution and 2 mL of extract was treated with 1 mL of lead acetate solution and white color was observed for the presence of flavonoids [25]. Followed by a ferric chloride test, 0.5 mL of extract was dissolved in 1 mL of water, mixed uniformly, and then two drops of ferric chloride solution were added and blue color was observed for the presence of gallic tannin. In the catecholic tannin test, test tubes contain 0.5 mL of extracts dissolved and uniformly mixed with 1 mL of water, and then, two drops of ferric chloride solution were added and green–black color was observed for the presence of catecholic tannin [23]. For the saponin froth test, 2.5 mL extract was added to 10 mL of sterile distilled water in a test tube and shaken vigorously for about 30 s. Honeycomb froth indicated the presence of saponins [23].

### 2.5. Quantitative Analysis of Phytochemical Constituent

#### 2.5.1. Total Phenolic Contents (TPCs)

The TPC was measured using gallic acid as a standard [26] with slight modifications. To prepare the gallic acid standard solution, 10 mg of pure gallic acid was dissolved in 80 mL of distilled water. The final volume was adjusted to 100 mL with distilled water to achieve a concentration of 0.1 mg/mL. Serial dilutions were then performed to obtain gallic acid concentrations of 12.5, 25, 50, 75, and 100 *μ*g/mL. For the blank solution, 0.5 mL of Folin–Ciocâlteu reagent (FCR), 1 mL of 7.5% Na_2_CO_3_, and 5.5 mL of distilled water were mixed. The 2N FCR reagent was diluted in a 1:10 ratio with distilled water before use. The reaction mixture for TPC determination was prepared by mixing 0.5 mL of diluted FCR reagent, 1 mL of the plant extract or standard gallic acid solution, 1 mL of 7.5% Na_2_CO_3_ (added after 3 min), and 4.5 mL of distilled water. The mixture was allowed to react at room temperature for 20 min. The TPC was then calculated using a standard curve generated from the gallic acid concentrations. The absorbance was recorded at 760 nm in a UV spectrophotometer (UV-1800, Shimadzu, Japan) against the reagent blank. Finally, the content of total phenolic compounds was determined using a reference curve with gallic acid.

#### 2.5.2. Total Flavonoid Contents (TFCs)

The TFC of the tea leaf and agarwood leaf methanol, ethanol, and chloroform extracts was estimated by the aluminum chloride assay [27]. A quercetin (QA) standard calibration curve was generated by preparing QA solutions of varying concentrations, 10 mg of QA was dissolved in 100 mL of methanol, and serial dilutions were performed to obtain concentrations of 12.5, 25, 50, 75, and 100 *μ*g/mL. To perform the assay, 1 mL of each plant extract or QA standard solution was combined with 3 mL of methanol in separate test tubes. Then, 0.2 mL of 10% aluminum chloride solution and 0.2 mL of 1 M potassium acetate solution were added, followed by 5.6 mL of distilled water, thoroughly mixed afterwards. After filtering all the prepared solutions through Whatman No. 1 filter paper, their absorbance was measured. A sample blank was prepared by substituting the aluminum chloride solution with distilled water. All solutions were incubated at room temperature for 30 min to ensure the reaction was complete. The intensity of the resulting yellow color was measured at 420 nm using a spectrophotometer. The absorbance values were then plotted against the concentration to create the calibration curve.

#### 2.5.3. Antioxidant Activity Analysis

##### 2.5.3.1. DPPH (2,2-Diphenyl-1-picrylhydrazyl) Scavenging Assay

Active antioxidant ability of plant extracts was determined by virtue of DPPH free radical scavenging assay as the method described by [28]. DPPH becomes colorless or pale yellow when neutralized by the chemical reaction. The readiness of DPPH solution and preparation of standard ascorbic acid: 4.0 mg dark violet–colored DPPH powder was dissolved in 100 mL of 95% methanol to prepare 0.004% (*w*/*v*) deep violet DPPH solution which was kept in a dark condition at room temperature. Varying concentration (25, 50, 75, and 100 *μ*g/mL) of ascorbic acid solution was prepared from the stock solution of 0.1 mg/mL concentration in methanol. Preparation of leaf extract and control: 5 mg of dry leaf powder was vortexed for 20 min in 10 mL methanol to make 0.5 mg/mL concentration and left at room temperature for 48 h (previously, we found having significant amount of flavonoids in methanol extracts and antioxidant activity has a proportional relationship to flavonoid content [29, 30], so we selected methanol instead of ethanol or chloroform). Filtrated extracts were used for serial dilution to prepare varying concentration (25, 50, 75, and 100 *μ*g/mL) of the solution, where 3 mL DPPH and 1 mL methanol solution were used as control.

##### 2.5.3.2. Procedure of DPPH Radical Scavenging Activity

One milliliter of each extract or standard at various concentrations (100, 75, 50, and 25 *μ*g/mL) was added to 3 mL of freshly prepared DPPH solution (0.004%) in methanol and mixtures were allowed to stand for 30 min in a dark place, and absorbance was recorded at 520 nm. The degree of decolorization of DPPH is proportional to the scavenging efficiency of the extract. Free radical scavenging activity of the methanol extracts of the plant sample, based on the scavenging activity of the stable free radical DPPH, was determined [28]. In this method, the activity of free radical scavenging extract or fractions is determined by measuring the intensity of the purple color from the DPPH methanol solution [31]. The percentage of inhibition of DPPH radical was calculated from the equation DPPH scavenging effect (%) = [(*A*_control_ − *A*_sample_)/*A*_control_ × 100].

DPPH scavenging activity was expressed graphically by plotting the absorbance data (percentage of inhibition of DPPH radical) against the concentration using the slope of the nonlinear regression. Inhibition concentration (IC_50_) is the concentration of an antioxidant that can cause 50% of DPPH to lose its radical characteristic which indicates the IC_50_ value is inversely proportional to the potential of free radical reduction. The greater the IC_50_ value gained, the lesser the potential for antioxidant activity. To compare leaf extract antioxidant activity, free radical scavenging activity experiment was conducted with slight modification [32].

### 2.6. Antimicrobial Assays and Synergistic Antimicrobial Assays of Leaf Extracts

Tea and agarwood leaves (5 g powder/80 mL methanol) with solvents were kept for 7 days at room temperature, and extracts were applied for antimicrobial assays by disc diffusion assay against the bacteria and fungus. Determination of the sensitivity of the strain to antibiotics was applied against the microbes. The screening of antimicrobial activity of the leaf extracts was carried out with agar disc diffusion method using Mueller–Hinton agar (MHA) medium [33, 34]. Bacterial and fungal culture (50 *μ*L) was taken from the nutrient broth culture and poured into the sterile plate containing MHA medium. Sterile cotton was used for streaking the dried surface of plates. Under aseptic condition, prepared 6-mm round filter paper discs and soaked with 50 *μ*L extract solution at a concentration of 1 mg/mL were air dried, placed into the center of an agar plate by using sterile forceps, and pressed down. Both 50 *μ*L of each type of leaf extracts were examined against 50 *μ*L of each bacterial and fungal culture. Under aseptic conditions, prepared discs containing extracts were placed in the agar plate by using sterile forceps and pressed down. The plates were then inverted and incubated at 37°C for 24 h. After incubation, each plate was examined. There was a circular zone of inhibition on the surface. The diameter of the complete zone of inhibition (judged by unaided eye) was measured including the diameter of the discs. Zones were measured to the nearest whole millimeter, using a ruler. All tests were performed in a triplicate manner.

In synergistic activity, 50 *μ*L (conc. of 1 mg/mL) of each type of leaf extracts was poured over the commercial antibiotics to evaluate their synergism, additive, or antagonism activity against the multidrug-resistant pathogens. Among the pathogen, three microbes, Gram-positive and Gram-negative bacteria and spore-forming fungus, were selected and only methanol leaf extracts were chosen as those indicate remarkable outcomes in phytochemical and antimicrobial analysis. To determine the synergistic antimicrobial activity, the bacterial strain was spread with a turbidity of 0.5 McFarland on MHA plates. Due to the assessments of the synergistic effects, selected antibiotic discs were discretely impregnated with 50 *μ*L of tea and agarwood leaf extracts spread over the antibiotics: E, Gen, Van, CTR, and Amx for antimicrobial activity analysis were evaluated against the bacteria and fungus. Antibiotic discs containing extracts were employed on the inoculated agar plates, and discs were anaerobically kept at 37°C for 24 h. After overnight incubation, the zones of inhibition produced by the combination of leaf extracts with standard antibiotics were assessed [33, 35] and there are three interpretations, namely, synergism, additive, and antagonism, whereas synergism was interpreted as if zones of combination treatment > zone of plant leaf extract + zone of the corresponding antibiotic, additive was interpreted as if the zone of combination treatment = zone of plant leaf extract + zone of correspondence antibiotic, and finally, antagonism was interpreted as if the zone of combination treatment < zone of plant leaf extract + zone of the corresponding antibiotic.

### 2.7. Statistical Analysis

Statistical analysis was done by STAT 10.0 for ANOVA. Antimicrobial activities and quantitative phytochemical experiment results were expressed as means ± standard deviation (SD). Differences were significant at the level of *p* < 0.01. The differences among groups in the tests were analyzed by two-way ANOVA and Tukey's test. Antioxidant scavenging activity and synergistic activity data analysis was done by Microsoft Excel 2013.

## 3. Result

### 3.1. Phytochemical Screening

#### 3.1.1. Qualitative Phytochemical Analysis

Qualitative analysis carried out on each plant leaf extract and the detection of alkaloids, tannins, flavonoid, steroid, and terpenoids indicate that these were major secondary metabolites as shown in Table 1. It shows that tannins, flavonoids, terpenoids, alkaloids, and glycosides were present in all the plant leaves. There was no steroid indication in this qualitative test, and in BT-6 and BT-7 leaf extracts, saponin was also absent.

#### 3.1.2. Quantitative Phytochemical Analysis

Both TPC and TFC of tea and agarwood leaf extracts were determined and expressed as microgram gallic acid equivalent (GAE)/milligram and microgram QA/milligram of sample, respectively. Data were listed according to leaf extracts.

##### 3.1.2.1. TPC

**Figure 1:**
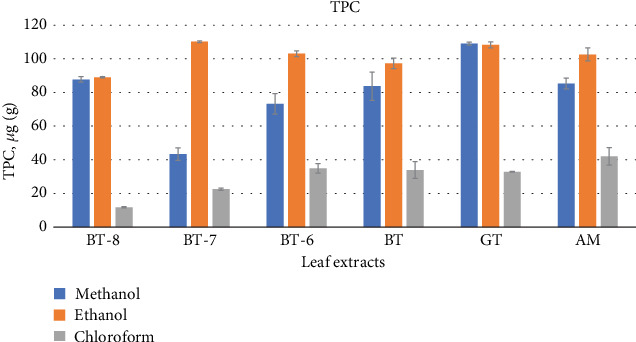
Graphical representation of TPC (total phenolic content) (mean ± SD) of six leaf extracts: BT-8, BT-7, BT-6, BT, GT, and agarwood leaves (AM) (*Aquilaria malaccensis*).

The results showed that the content of total phenols in extracts, expressed as GAEs of plant leaf, varied to a great extent and ranged from 43.35 *μ*g GAE/mg (BT-7) to 109.06 *μ*g GAE/mg (GT) in methanol extracts and 89.03 *μ*g GAE/mg (BT-8) to 110.16 *μ*g GAE/mg (BT-7) in ethanol extracts as well as in chloroform extracts 10.0 *μ*g (BT-8) to 42.0 *μ*g GAE/mg (agarwood leaves) (Figure 1).

##### 3.1.2.2. TFC

**Figure 2:**
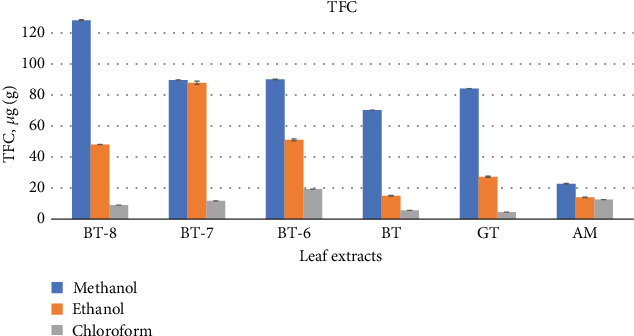
Graphical representation of TFC (total flavonoid content) (mean ± SD) of six leaf extracts BT-8, BT-7, BT-6, BT, GT, and agarwood leaves (AM) (*Aquilaria malaccensis*).

The difference in TFC among studied plant leaf extracts varied significantly; in methanol extracts, it ranges from 22.68 *μ*g QA/mg (agarwood leaves) to 128.1 *μ*g QA/mg (BT-8); nevertheless, all fresh teas—BT-8, BT-6, and BT-7—and GT showed the maximum result. In ethanol extracts, it ranged from 13.99 (agarwood leaves) to 51.12 *μ*g QA/mg (BT-6), and in chloroform extracts, it ranged from 4.51 (GT) to 19.29 *μ*g QA/mg (agarwood leaves) (Figure 2).

##### 3.1.2.3. Antioxidant Activity

**Figure 3:**
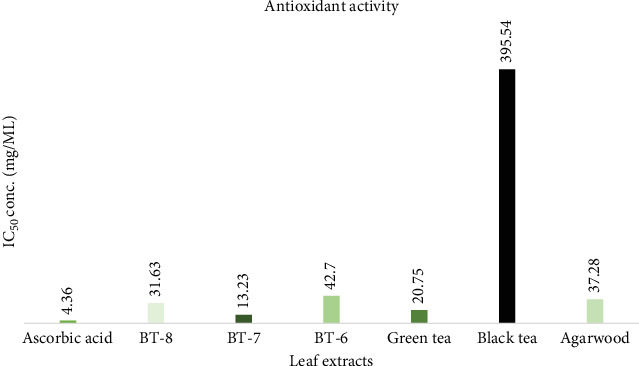
IC_50_ values of tea and agarwood leaf extracts.

Tea leaf extracts from BT-6, BT-7, BT-8, GT, and BT and agarwood leaves in methanol were analyzed for an antioxidant activity where ascorbic acid was the control. Concentrations of each extract able to yield an absorbance value of 0.5 were determined from the graph of absorbance at 517 nm against extract concentrations (Supporting Information 4: File S1). Substances that have high antioxidant activity will have a low IC_50_ value. The IC_50_ values of all leaf extracts are where standard ascorbic acid contains 4.366 mg/mL. According to the standard ascorbic acid value, the leaf extracts contain the serial IC_50_ value of ascorbic acid < BT‐7 < GT < BT‐8 < AM < BT‐6 < BT (Figure 3).

### 3.2. Antimicrobial Activity

#### 3.2.1. Determination of the Strain's Sensitivity to Antibiotics

A total of five antibiotics were used for multidrug resistance analysis against the six pathogens. According to the CLSI standard, most of the Gram-negative bacteria were resistant to the selected antibiotics. *S. aureus* and *Mucor circinelloides* also showed resistance and intermediary results against the antibiotics (Supporting Information 1: Table S1).

#### 3.2.2. Antimicrobial Susceptibility of Leaf Extracts

**Figure 4:**
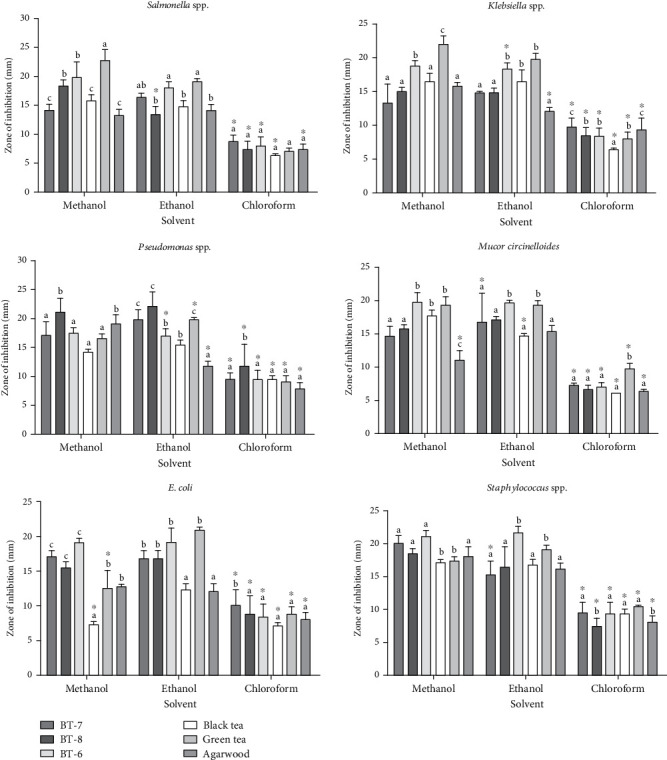
Antimicrobial activity of leaves (BT-7, BT-8, BT-6, black tea, green tea, and agarwood) in methanol, ethanol, and chloroform extracts against *Salmonella* spp., *Pseudomonas aeruginosa*, *Klebsiella pneumoniae*, *Mucor circinelloides, E. coli*, and *Staphylococcus aureus*.

Leaf extracts in methanol, ethanol, and chloroform were analyzed for their antimicrobial activity against bacteria and fungus. These results were observed among Gram-positive and Gram-negative bacteria and spore-forming fungus in all types of extracts (Figure 4). In methanol, GT extracts showed 22.6 ± 1.8 mm and 22.0 ± 1.1 mm highest zone of inhibition against *Salmonella* spp. and *K. pneumoniae*. In ethanol, BT-6 extracts showed 21.6 ± 0.8 mm highest zone of inhibition against *S. aureus* where GT showed 19 mm zone of inhibition against all microbes. In methanol, all leaf extracts showed excellent antimicrobial activity against the microbes. In ethanol, fresh tea BT-6 and BT-8 and GT extracts showed remarkable activity against the microbes. In chloroform, all leaf extracts showed less significant results compared to the extracts in methanol and ethanol against the microbes. Here, data are represented as mean ± SD. Means within a row with a different superscript differ significantly (*p* < 0.05) (Figure 4). All BT-8, BT-7, BT-6, BT, GT, and agarwood leaf extracts showed significant (*p* < 0.05) value against the microbes except *P. aeruginosa* and *S. aureus* in methanol extracts and *Mucor circinelloides* and *S. aureus* in ethanol extracts while most of the values are nonsignificant in chloroform extracts. Comparing the activity between antibiotics and leaf extracts in Supporting Information 1: Table S1 and Figure 4, data can be described as follows: (a) Ethanol extracts of BT-6 showed 19 mm diameter of zone of inhibition against *E. coli* whereas Gen showed 18 mm. (b) Methanol extracts of BT-8 showed 18 mm against *S. aureus* where CTR showed 12 mm diameter zone of inhibition. (c) Ethanol extracts of BT-7 signified 19 mm against *P. aeruginosa* whereas Van showed only 8 mm zone of inhibition against that bacteria. (d) Methanol extracts of BT-6 showed 19 mm against *Mucor* while E showed 15 mm zone of inhibition against this fungus. So, these results indicate that leaf extracts have noticeable antimicrobial activity than commercial antibiotics.

### 3.3. Synergistic Activity of Leaf Extracts With Antibiotics Against Microbes

Synergistic activity of mentioned plant leaf extracts spreading over the selected antibiotics was evaluated by measuring the diameter of the zone of inhibition method (Tables 2, 3, and 4 and Supporting Information 3: Figure S2). Leaf extracts were spread over the antibiotics and antimicrobial activity against *E. coli*, *Mucor circinelloides*, and *S. aureus.* The combination of BT-8 with CTR showed a strong synergistic outcome (36 mm) against *E. coli* bacteria. Both GT and agarwood leaf extracts in combination with E (33 mm), agarwood leaf extracts with Van (23 mm), and BT-8 with Van (22 mm) showed synergistic activity against this pathogenic *E. coli* bacteria (Table 2). However, both BT and BT-7 showed antagonism results in this case. Except BT, most of the extracts combined with antibiotics showed the activity against *Mucor circinelloides*. Both GT with Gen and agarwood leaf extracts with CTR showed strong synergistic activity (35 mm) against this pathogenic fungus. Additionally, Van, E, and even Amx (already listed as resistant by microbes) showed synergism with tea and agarwood leaf extracts against the fungus whereas Van with BT-6 (31 mm) and BT-8 (27 mm) and GT (27 mm) showed synergistic activity. Moreover, BT-7 with CTR (32 mm) and Amx (22 mm) against and E with agarwood leaves (28 mm) indicated synergism outcomes while both BT and BT-6 showed antagonism results against this pathogenic *Mucor circinelloides* fungus (Table 3). No synergetic effect was observed when different antibiotics were used with extracts from BT, agarwood leaves, BT-6, and BT-8 against *S. aureus* (Table 4). However, BT and GT leaf extracts with E showed additive results (36 mm) against this pathogenic bacterium. Additive results are also noticed in BT-7 with CTR against *E. coli* (36 mm) and BT-8 with E (30 mm) against *Mucor circinelloides* (Tables 2 and 3).

## 4. Discussion

### 4.1. Phytochemical Analysis

All six leaf extracts from tea and agarwood showed different phytochemical constituents depending on their variety, type, and extraction procedure in both qualitative and quantitative phytochemical analysis. In the qualitative analysis of the extracts, steroids were not present but alkaloids, flavonoids, terpenoids, tannin, glycosides, and saponins were found. Some authors have also found these bioactive compounds except alkaloids in agarwood plant leaves [36]. Similarly, the quantity and makeup of polyphenols in tea plants might vary depending on the type of tea, how it is harvested, how it is processed, and other factors [37]. Both BT-7 and GT contained the best results in TPC. Though the quantity from BT-8 and BT-6 was not insignificant, BT-7 extracts demonstrated the highest 110.16 *μ*g GAE/mg result in ethanol extracts and GT provided 109.10 *μ*g GAE/mg in methanol extracts. This data can be compared to the findings of other researchers' studies where they presented that fresh tea leaves and commercial GT both had 105 mg/mL and 86 mg/mL of polyphenols, respectively [33]. With the exception of agarwood leaves, all extracts showed significant TFC in methanol extracts where BT-8 tea leaves showed highest (128.1 *μ*g QA/mg) followed by BT-6 (90 *μ*g QA/mg) and BT-7 (89.64 *μ*g QA/mg) tea leaves and GT (84.07 *μ*g QA/mg) in methanolic extracts while extracts both in ethanol (except BT-7) and chloroform presented comparably lower amount of TFC. These leaf extract TPC and TFC data can compete other medicinal plant phytochemicals too [38]. In other research, authors have found aqueous GT extracts had 107.9 *μ*g QA/mg TFC [39] which is higher than methanol GT extracts but lower than fresh tea BT-8.

### 4.2. Antioxidant Activity

The IC_50_ (which is inversely proportional to antioxidant activity) value of BT-7, GT, and BT-8 showed significant values 13.23 mg/mL, 20.75 mg/mL, and 31.63 mg/mL, respectively, which mean higher antioxidant activity, and this indicates that the high amount of TPC and TFC content was related to high antioxidant activity. Corresponding to author result showed high flavonoid content (flavan-3-ols) is responsible for the highest antioxidant activity in GT [40]. Besides, agarwood leaves and BT contain 37.28 and 395.54 mg/mL accordingly. Some researchers have identified Senggani (*Melastoma candidum* D. Don) leaves have a very powerful antioxidant activity; in methanol and chloroform extract, the IC_50_ values were 65,6521 and 43,8924 *μ*g/mL, respectively [32], while in this study data from all extracts including BT 395.54 mg/mL indicates higher antioxidant activity than Senggani. GT contains more antioxidant activity than BT and supporting this study results have found the same in other research [34]. The authors have determined the IC_50_ value from agarwood leaves about 27.88 ppm [36].

### 4.3. Antimicrobial Activity

The evaluation of antimicrobial activity was based on the measurement of the diameter of the inhibition zones formed around the discs. Here, only five antibiotics from different class and generation were applied against the pathogens while methanol and ethanol leaf extract especially fresh tea and GT presented potential antimicrobial activities against the multidrug-resistant pathogen. In this study, we choose the same antibiotics both for bacteria and fungus aiming to get uniform result and comparison. Among the solvent extractions, methanol and ethanol extracts showed higher antimicrobial activity than chloroform extracts (Figure 4 and Supporting Information 2: Figure S1). Supporting this result, some research presented that the type of solvent for extraction of the bioactive compounds of the plants may have an impact on antimicrobial activity [41, 42]. Extracts against pathogens were subjected three times, and the average zone of inhibition values were higher than the commercial antibiotics. According to the zone of inhibition, both methanol and ethanol extracts of BT-6 and BT-7 showed significant results against *P. aeruginosa* and *E. coli*, respectively. However, chloroform leaf extracts had poor results against the pathogens. However, authors reported that tea extracts had selective antibacterial activity based upon the type of the extracts, concentration, and bacterial species [10, 43]. Reports suggested that pathogenic microorganisms such as *E. coli*, *Enterococcus faecalis*, *S. aureus*, *Candida albicans*, and *P. aeruginosa* are sensitive to fresh tea extracts [33]. GT ethanol extracts showed a consistency remarkable result (19 mm) against all the bacteria including Gram-positive and Gram-negative bacteria and fungus. Similarly, GT extracts from methanol inhibited the most against *Salmonella* spp. and *K. pneumoniae* around 22 mm where third-generation Gen showed lower than 20 mm against all selected Gram-negative bacteria and fungus. Another study mentioned that the zone of inhibition of GT was listed 17.55 ± 0.393 mm against *P. aeruginosa* ATCC 27853 and 18.970 ± 0.287 mm against *S. aureus* ATCC 25923 [44]. Fresh tea BT-8 showed highest against *P. aeruginosa*, and GT showed highest against antimicrobial activity of *Salmonella* spp. and *K. pneumoniae*. The reason behind the antibacterial action of tea such as GT catechins is that polyphenols have the ability to bind to the bacterial lipid bilayer cell membrane which promotes to damage the membrane [10].

Nevertheless, BT extract activities against *S. aureus* and *Mucor circinelloides* presented maximum of 17 mm zone of inhibition while GT presented stronger activity against Gram-negative and Gram-positive bacteria as well as fungus. In support of this result, compared to BT, stronger antimicrobial activity from GT extracts was reported against Gram-positive and Gram-negative bacteria [45]. Compared to synthetic standard antibiotics (Supporting Information 1: Table S1), leaf extracts indicated a remarkable difference in zone of inhibition. Maximum leaf extracts showed lower inhibition zone against Gram-negative *E. coli* than Gram-positive *S. aureus*, and some researchers identified that different tea extracts had antimicrobial activity against *S. aureus* and *B. subtilis*, while *E. coli* (Gram-negative bacteria) were resistant to the extracts [46]. In general, Gram-negative bacteria are more resistant to polyphenols due to their diverse composition of the cell wall [47]. Some authors also showed comparison between their synthesized compounds (thiazolo pyrazole and thiazolo pyridine) with Gen and ketoconazole as standard drugs where they found that compounds are the most active displaying good inhibitory potency against *S. epidermidis*, *B. subtilis*, *S. pneumoniae*, and *K. pneumoniae* higher than the correspondent drugs [48]. Another research identified chemical composition of the methanol (80%) extract of *Pithecellobium dulce* seed (Hail, Saudi Arabia) has the ability to inhibit the growth of multidrug-resistant bacteria, where extracts showed antibacterial activity toward tested clinical bacterial strains with MIC values ranging from 233 mg/mL for *Acinetobacter baumannii* to 300 mg/mL for *S. aureus* and *E. coli* [49].

In this antimicrobial activity analysis, plant leaves against the microbes were found to be effective in GT leaves followed by fresh tea leaves, agarwood leaves, and BT. The leaves with the highest levels of phenolic and flavonoid content and robust antioxidant activity indicated the highest antibacterial activity.

### 4.4. Synergetic Activity

The extract effects on various antibiotics including resistance drug synergistic antimicrobial activities were analyzed (Tables 2, 3, and 4 and Supporting Information 3: Figure S2). Understanding a new method for the adjunctive treatment of microbial infections may be made easier by considering the synergistic effects of combination therapy. In this research, both fresh tea BT-7 and BT-8 (combined with CTR) indicated the highest zone 36 mm against *E. coli* and both GT (combined with Gen) and agarwood leaves (combined with CTR) showed 35 mm against *Mucor* fungus. A relevant research claimed, synergistic activity combined with fresh tea, GT with chloramphenicol against not only pathogenic bacteria but fungus as well [33]. The authors have also found the synergism antimicrobial activity of Gen and E against bacteria and fungus [50, 51]. Interestingly, *Mucor* fungus showed more susceptibility against E than Gen where the gap is 2 mm zone of inhibition in both antibiotic and synergistic assays. The reason is not clear but the unique properties of this fungus described by some authors [52, 53]. In case of GT, similar research showed that broad range of antibiotics chloramphenicol, Amx, cotrimoxazole, azithromycin, levofloxacin, Gen, methicillin, nalidixic acid, and ciprofloxacin with GT extracts had synergistic activity against *E. coli* [43]. Reports also suggest that the highest synergistic activity (*C. zeylanicum* combined with Amx) was 38 mm against *E. coli* [54]. However, no synergistic action against *S. aureus* was found in this study. In synergistic activity fresh tea, GT and agarwood leaf extracts showed synergism against drug-resistant pathogens whereas *E. coli* and *Mucor* fungus were found more sensitive than *S. aureus*. This finding indicates that synergetic activity is possible against Gram-negative *E. coli* and spore-forming *Mucor* fungus but Gram-positive *S. aureus* is still a challenge where cell wall composition could be a reason was explained [46]. The authors have also found synergism antimicrobial activity of Gen and E against bacteria and fungus [50, 51].

After analyzing antimicrobial activity, there were found close relations between phytochemical constituency and antimicrobial activity. In this study, fresh tea varieties such as BT-7, BT-8, and GT contain prominent antimicrobial activity against all selected pathogenic bacteria and fungus; nevertheless, other leaves' activity was not negligible. Data indicates TPC and TFC are related to antioxidant activity and finally antimicrobial potency. Though in the past the development of resistant strains was directly correlated with the production of new antibiotics, the current mainstream strategy for combating diseases is focused on the modification and alteration of existing antibiotics to combat emerging and re-emerging pathogen resistance globally [55]. Due to having potential therapeutic compounds, tea and agarwood plant leaves may act as a blessing in that case. Besides these leaves containing health-beneficial bioactive compounds, people may get rid of the pathogenic infection and gain sound health by consuming them as a drink. Results suggest that tea leaves have remarkable antioxidant activity as well as phytochemical constitute close effect on antimicrobial activity against pathogens. Therefore, natural herbal therapy after commercial production can create a new era in pharmaceutical industry as well as combat against antibiotic resistance. Identification of phytochemicals is important to analyze the medicinal properties of the bioactive compounds, and controlling the quality and standardization of extracts is required for therapeutic preparations from natural sources. This study highly encourages researchers to conduct HPLC analysis or GC-Mass to identify the most common bioactive molecules in the future which can be the most promising result in tea and agarwood in Bangladesh, and tea variety information and research are readily available in Bangladesh Tea Research Institute [56]. However, the research limitations of this study can be overcome by extending and considering broad spectrum analysis such as standardization as well as determination of minimum inhibition concentration (MIC) of the extracts for antimicrobial activity and, moreover, synergistic activity; wide-ranging types of solvents such as aqueous, acetone, or other polar or nonpolar type for extraction; broad ranges of antibiotic application; and comparison with leaf extracts in the future. Indeed, more research development and innovations may be fruitful in this area of blooming natural or bioantibiotics instead of synthetic antibiotics.

## 5. Conclusions

In the global emergence of multidrug-resistant microbes and decline of effective antimicrobial drugs, tea and agarwood leaf extracts as antibiotic compounds can be an alternative to synthetic antibiotics. Fresh tea and GT showed maximum TPC, TFC, antioxidant activity, and antimicrobial activity against multidrug-resistant bacteria and fungus. Besides, some effective synergistic activities are also noticed. However, numerous types of results were obtained on the basis of leaf type, leaf extracts, microbial complex structure, etc. Considering all the factors, data, and parameters, it may be concluded that after further research on standardization of tea and agarwood leaf extracts antibiotic compounds will have the potential to fight against antibiotic resistance and boost up the immunity.

## Figures and Tables

**Table 1:** Qualitative phytochemical analysis of BT-8, BT-7, BT-6, black tea (BT), green tea (GT), and agarwood leaf (AM) extracts.

**Phytochemicals**	**Samples**					
	**BT-6**	**BT-7**	**BT-8**	**AM**	**BT**	**GT**
Alkaloid	**+++**	**++**	**+**	**++**	**+**	**++**
Tannin	**+**	**+**	**+**	**+**	**++**	**+++**
Saponin	**−**	**−**	**+**	**+**	**+++**	**++**
Flavonoid	**+**	**+**	**+**	**+**	**++**	**+++**
Terpenoid	**+**	**+++**	**+++**	**+**	**++**	**+**
Steroid	**−**	**−**	**−**	**−**	**−**	**−**
Glycoside	**++**	**++**	**+**	**+**	**+**	**+**

*Note:* +++ = appreciable amount, ++ = moderate amount, + = trace amount, **−** = not detected.

**Table 2:** The synergistic activity of leaf extracts with antibiotics against *E. coli.*

***E. coli***					
**Sample name**	**Antibiotics**	**Inhibition zone diameter (mm)**	**Equation**	**Combined inhibition zone diameter (mm)**	**Outcome**
Black tea	Van	17 + 7	>	22	Antagonism
	Gen	17 + 18	>	25	Antagonism
	E	17 + 17	>	30	Antagonism
	Amx	17 + 6	>	10	Antagonism
	CTR	17 + 20	>	36	Antagonism

Green tea	Van	7 + 15	>	17	Antagonism
	Gen	18 + 15	>	22	Antagonism
	E	17 + 15	<	33	Synergism
	Amx	6 + 15	>	6	Antagonism
	CTR	20 + 15	>	13	Antagonism

Agarwood leaves	Van	12 + 7	<	23	Synergism
	Gen	12 + 18	>	27	Antagonism
	E	12 + 17	<	33	Synergism
	Amx	12 + 6	>	12	Antagonism
	CTR	12 + 19	>	18	Antagonism

BT-6	Van	19 + 7	>	15	Antagonism
	Gen	19 + 18	>	23	Antagonism
	E	19 + 17	>	32	Antagonism
	Amx	19 + 6	>	6	Antagonism
	CTR	19 + 19	>	14	Antagonism

BT-8	Van	13 + 7	<	22	Synergism
	Gen	13 + 18	>	24	Antagonism
	E	13 + 17	>	28	Antagonism
	Amx	13 + 6	>	6	Antagonism
	CTR	13 + 19	<	36	Synergism

BT-7	Van	17 + 7	>	22	Antagonism
	Gen	17 + 18	>	25	Antagonism
	E	17 + 17	>	30	Antagonism
	Amx	17 + 6	>	10	Antagonism
	CTR	17 + 19	=	36	Additive

**Table 3:** The synergistic activity of leaf extracts with antibiotics against *Mucor circinelloides.*

***Mucor circinelloides***					
**Sample name**	**Antibiotics**	**Inhibition zone diameter (mm)**	**Equation**	**Combined inhibition zone diameter (mm)**	**Outcome**
Black tea	Van	6 + 17	>	20	Antagonism
	Gen	13 + 17	>	22	Antagonism
	E	15 + 17	>	18	Antagonism
	Amx	6 + 17	>	17	Antagonism
	CTR	17 + 17	>	28	Antagonism

Green tea	Van	6 + 20	<	27	Synergism
	Gen	13 + 20	<	35	Synergism
	E	15 + 20	>	25	Antagonism
	Amx	6 + 20	>	20	Antagonism
	CTR	17 + 20	>	22	Antagonism

Agarwood leaves	Van	6 + 11	>	29	Antagonism
	Gen	13 + 11	<	30	Synergism
	E	15 + 11	<	28	Synergism
	Amx	6 + 11	>	15	Antagonism
	CTR	17 + 11	<	35	Synergism

BT-6	Van	6 + 21	<	31	Synergism
	Gen	13 + 21	>	25	Antagonism
	E	15 + 21	>	30	Antagonism
	Amx	6 + 21	>	15	Antagonism
	CTR	17 + 21	>	28	Antagonism

BT-8	Van	6 + 15	<	27	Synergism
	Gen	13 + 15	>	24	Antagonism
	E	15 + 15	=	30	Additive
	Amx	6 + 15	>	15	Antagonism
	CTR	17 + 15	>	25	Antagonism

BT-7	Van	6 + 14	<	31	Synergism
	Gen	13 + 14	<	30	Synergism
	E	15 + 14	>	27	Antagonism
	Amx	6 + 14	<	22	Synergism
	CTR	17 + 14	<	32	Synergism

**Table 4:** The synergistic activity of leaf extracts with antibiotics against *Staphylococcus aureus.*

***Staphylococcus aureus***					
**Sample name**	**Antibiotics**	**Inhibition zone diameter (mm)**	**Equation**	**Combined inhibition zone diameter (mm)**	**Outcome**
Black tea	Van	24 + 16	>	25	Antagonism
	Gen	16 + 20	>	26	Antagonism
	E	16 + 20	=	36	Additive
	Amx	16 + 6	>	12	Antagonism
	CTR	16 + 12	>	11	Antagonism

Green tea	Van	24 + 17	>	27	Antagonism
	Gen	17 + 20	>	25	Antagonism
	E	17 + 19	=	36	Additive
	Amx	17 + 6	>	15	Antagonism
	CTR	17 + 12	>	12	Antagonism

Agarwood leaves	Van	24 + 18	>	26	Antagonism
	Gen	18 + 20	>	28	Antagonism
	E	18 + 22	>	33	Antagonism
	Amx	18 + 6	>	7	Antagonism
	CTR	18 + 12	>	13	Antagonism

BT-6	Van	24 + 21	>	26	Antagonism
	Gen	21 + 20	>	26	Antagonism
	E	21 + 22	>	32	Antagonism
	Amx	21 + 6	>	15	Antagonism
	CTR	21 + 12	>	11	Antagonism

BT-8	Van	24 + 17	>	25	Antagonism
	Gen	17 + 20	>	25	Antagonism
	E	17 + 22	>	31	Antagonism
	Amx	17 + 6	>	6	Antagonism
	CTR	17 + 12	>	22	Antagonism

BT-7	Van	24 + 17	>	25	Antagonism
	Gen	17 + 20	>	18	Antagonism
	E	17 + 22	>	12	Antagonism
	Amx	17 + 6	>	22	Antagonism
	CTR	17 + 11	=	28	Additive

## Data Availability

The data can be available based on reasonable request and consent from all authors.

## Supporting Information

Supporting Information 1 Table S1: microbial sensitivity test to antibiotics.

Supporting Information 2 Figure S1: antimicrobial activity of leaf extracts. (a) BT-6 against *E. coli*. (b) BT-8 against *Staphylococcus aureus*. (c) BT-7 against *Pseudomonas aeruginosa*. (d) BT-6 against *Mucor circinelloides*.

Supporting Information 3 Figure S2: synergistic activity of leaf extracts with synthetic antibiotics: Van = vancomycin; Amx = amoxicillin; Gen = gentamicin; E = erythromycin; CTR = ceftiofur. (a) Green tea (GT) against *Mucor circinelloides*. (b) BT-6 against *E. coli*. (c) Black tea (BT) against *Staphylococcus aureus*. (d) BT-6 against *Staphylococcus aureus.*

Supporting Information 4 File S1: graph of absorbance at 517 nm against each type of extract concentrations.
