# Supplementary material for: Antimicrobial Activity of Tea and Agarwood Leaf Extracts Against Multidrug-Resistant Microbes
Source: Biomed Res Int. 2024 Dec 19;2024:5595575. doi: 10.1155/bmri/5595575 (PMC11671646; doi:10.1155/bmri/5595575)
Supplement: Supporting Information 1 — Table S1: microbial sensitivity test to antibiotics. [file 5595575.f1.docx]

Supplementary Table 1: Microbial sensitivity test to antibiotics

| Name of the | Zone of inhibition against microbes (mm) | | | | | |
| --- | --- | --- | --- | --- | --- | --- |
| Antibiotics | *E. coli* | *Pseudomonas spp.* | *Staphylococcus spp.* | *Klebsiella spp.* | *Salmonella spp.* | *Mucor*  circinelloides |
| Vancomycin | 7 | 8 | 23 | 7 | 8 | 6 |
| Gentamicin | 18 | 11 | 24 | 16 | 13 | 13 |
| Erythromycin | 17 | 12 | 25 | 9 | 17 | 15 |
| Ceftiofur | 19 | 18 | 12 | 14 | 19 | 17 |
| Amoxicillin | 6 | 6 | 7 | 6 | 6 | 6 |
