# Supplementary material for: Antimicrobial Activity of Tea and Agarwood Leaf Extracts Against Multidrug-Resistant Microbes
Source: Biomed Res Int. 2024 Dec 19;2024:5595575. doi: 10.1155/bmri/5595575 (PMC11671646; doi:10.1155/bmri/5595575)
Supplement: Supporting Information 2 — Figure S1: antimicrobial activity of leaf extracts. (a) BT-6 against E. coli. (b) BT-8 against Staphylococcus aureus. (c) BT-7 against Pseudomonas aeruginosa. (d) BT-6 against Mucor circinelloides. [file 5595575.f2.docx]

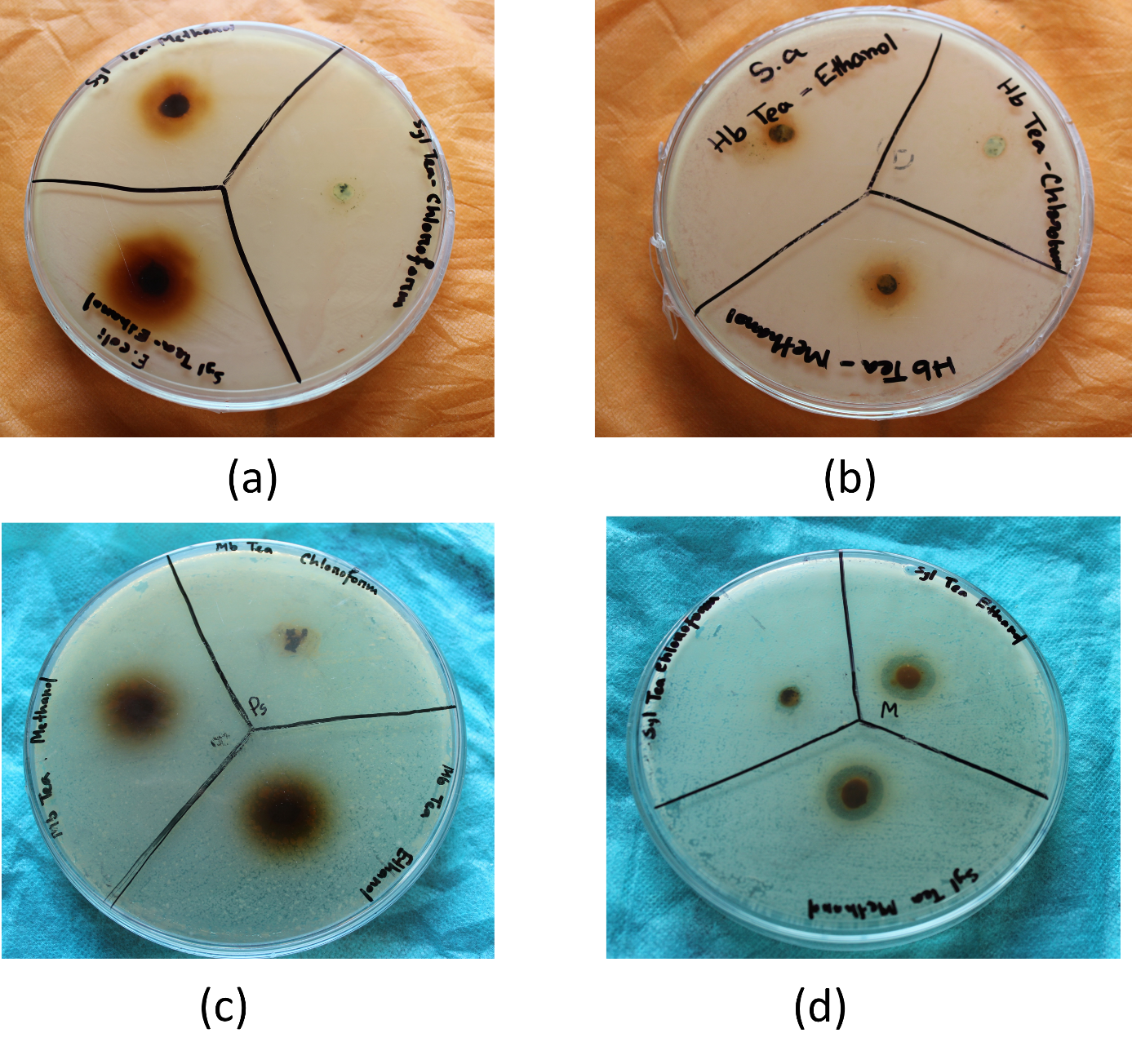


Supplementary Figure 1: Antimicrobial activity of leaves extracts (a) BT-6 against *E. coli* (b) BT-8 against *Staphylococcus aureus* (c) BT-7 against *Pseudomonas aeruginosa* and (d) BT-6 against *Mucor circinelloides.*
