# Supplementary material for: Antimicrobial Activity of Tea and Agarwood Leaf Extracts Against Multidrug-Resistant Microbes
Source: Biomed Res Int. 2024 Dec 19;2024:5595575. doi: 10.1155/bmri/5595575 (PMC11671646; doi:10.1155/bmri/5595575)
Supplement: Supporting Information 3 — Figure S2: synergistic activity of leaf extracts with synthetic antibiotics: Van = vancomycin; Amx = amoxicillin; Gen = gentamicin; E = erythromycin; CTR = ceftiofur. (a) Green tea (GT) against Mucor circinelloides. (b) BT-6 against E. coli. (c) Black tea (BT) against Staphylococcus aureus. (d) BT-6 against Staphylococcus aureus. [file 5595575.f3.docx]

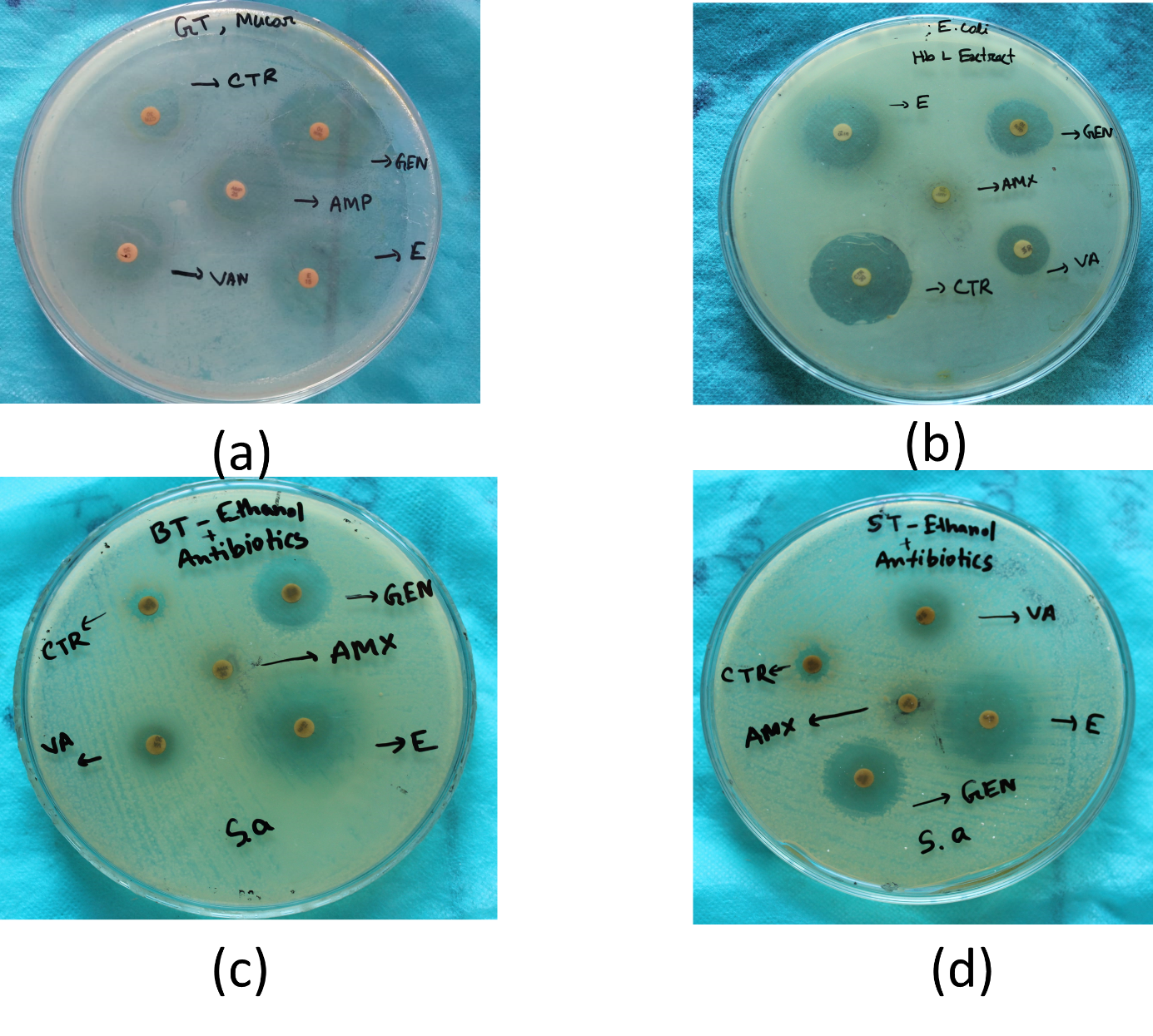


Supplementary Figure 2: Synergistic activity of leaves extracts with synthetic antibiotics: Van=Vancomycin; Amx=Amoxicillin; Gen=Gentamicin; E=Erythromycin and CTR= Ceftiofur. (a) Green Tea (GT) against *Mucor* (b) BT-6 against *E. coli* (c) Black Tea (BT) against *Staphylococcus aureus* (d) BT-6 against *Staphylococcus aureus.*
