# Supplementary figures and images for: Antimicrobial Activity of Tea and Agarwood Leaf Extracts Against Multidrug-Resistant Microbes
Source: Biomed Res Int. 2024 Dec 19;2024:5595575. doi: 10.1155/bmri/5595575 (PMC11671646; doi:10.1155/bmri/5595575)

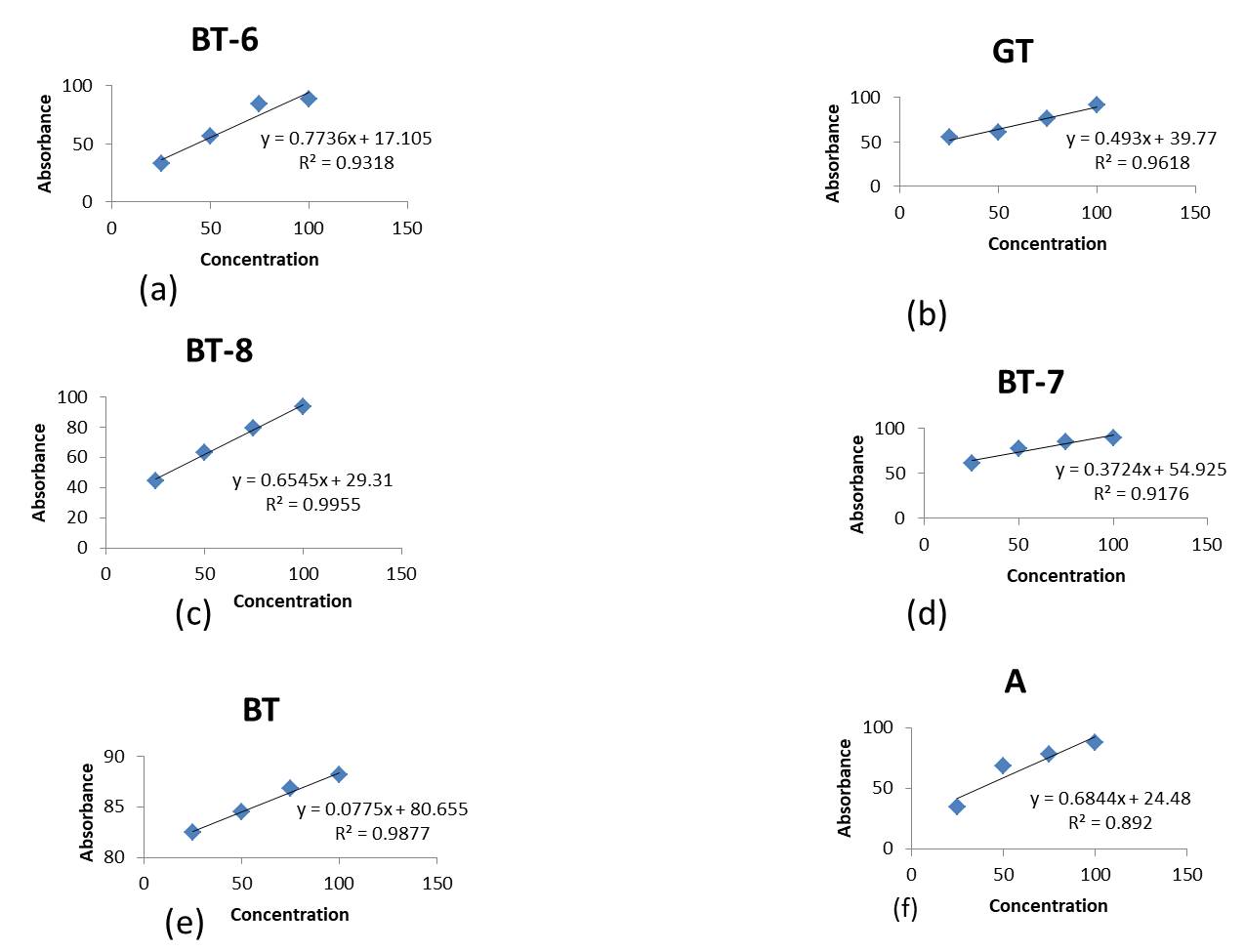


Supplementary file 1: Graph of absorbance at 517 nm against each type extracts concentrations

Supplement: Supporting Information 4 — File S1: graph of absorbance at 517 nm against each type of extract concentrations. [file 5595575.f4.docx]
